# Shikonin enhances efficacy of a gene-based cancer vaccine via induction of RANTES

**DOI:** 10.1186/1423-0127-19-42

**Published:** 2012-04-12

**Authors:** Hui-Ming Chen, Pi-Hsueh Wang, Kandan Aravindaram, Yun-Hsiang Chen, Hsiu-Hui Yu, Wen-Chin Yang, Ning-Sun Yang

**Affiliations:** 1 Department and Institute of Pharmacology, National Yang-Ming University, Taipei, Taiwan; 2 Institute of Agricultural Biotechnology Research Center, Academia Sinica, Taipei, Taiwan; 3 Institute of Biotechnology, National Taiwan University, Taipei, Taiwan; 4 Department of Life Sciences, National Central University, Taipei, Taiwan; 5 Graduate institute of biotechnology, National Chung Hsing University, Taichung, Taiwan; 6 Institute of Agricultural Biotechnology Research Center, Academia Sinica, No. 128, Academia Sinica Rd. Sec. 2, Nankang District, Taipei 11529, Taiwan

## Abstract

**Background:**

Shikonin, a phytochemical purified from *Lithospermum erythrorhizon*, has been shown to confer diverse pharmacological activities, including accelerating granuloma formation, wound healing, anti-inflammation and others, and is explored for immune-modifier activities for vaccination in this study. Transdermal gene-based vaccine is an attractive approach for delivery of DNA transgenes encoding specific tumor antigens to host skin tissues. Skin dendritic cells (DCs), a potent antigen-presenting cell type, is known to play a critical role in transmitting and orchestrating tumor antigen-specific immunities against cancers. The present study hence employs these various components for experimentation.

**Method:**

The mRNA and protein expression of RANTES were detected by RT-PCR and ELISA, respectively. The regional expression of RANTES and tissue damage in test skin were evaluated via immunohistochemistry assay. Fluorescein isothiocyanate sensitization assay was performed to trace the trafficking of DCs from the skin vaccination site to draining lymph nodes. Adjuvantic effect of shikonin on gene gun-delivered human gp100 (hgp100) DNA cancer vaccine was studied in a human gp100-transfected B16 (B16/hgp100) tumor model.

**Results:**

Among various phytochemicals tested, shikonin induced the highest level of expression of RANTES in normal skin tissues. In comparison, mouse RANTES cDNA gene transfection induced a higher level of mRANTES expression for a longer period, but caused more extensive skin damage. Topical application of shikonin onto the immunization site before gene gun-mediated vaccination augmented the population of skin DCs migrating into the draining lymph nodes. A hgp100 cDNA gene vaccination regimen with shikonin pretreatment as an adjuvant in a B16/hgp100 tumor model increased cytotoxic T lymphocyte activities in splenocytes and lymph node cells on target tumor cells.

**Conclusion:**

Together, our findings suggest that shikonin can effectively enhance anti-tumor potency of a gene-based cancer vaccine via the induction of RANTES expression at the skin immunization site.

## Background

Shikonin, a phytochemical from the traditional herbal medicine *Lithospermum erythrorhizon*, has been shown to possess diverse pharmacological properties, including anti-oxidant [1], wound healing [2-4], anti-inflammatory [5] and anti-tumor properties [6,7]. Anti-inflammatory activities of shikonin can be mediated by suppression of TNF-α promoter activity [8] or by regulation of TNF-α pre-mRNA splicing [9]. Wound-healing activity of shikonin resulted in proliferation of fibroblasts and collagen fiber levels in granuloma tissues, and an increase in the CD11b^+ ^cell population in granulation tissues [3,4]. These findings suggest that shikonin may confer cellular activities that can induce specific chemokines and subsequent chemotactic activities in specific immune-responsive cell types.

Transdermal or dermal delivery as a strategy of administration for specific vaccines, therapeutic agents and experimental medicines, is an attractive approach due to the presence and unique features of key immunocompetent cells in skin tissues, such as keratinocytes and dendritic cells (DCs), which include both dermal and epidermal DCs. DCs are potent professional antigen presenting cells that can act as sentinels that constantly monitor the surrounding milieu. They can be activated by a variety of cellular or microenvironmental signals produced by invading pathogens or neighboring cells [10,11]. Activated skin DCs can migrate from the epidermis or dermis to the draining lymph nodes where antigen presentation takes place [12]. On the basis of the specific characteristics of DCs, advanced vaccine strategies have been designed to target antigens onto DCs, promoting cross-priming and improving peptide-binding to both MHC class I and class II molecules [13]. Evidence has accumulated showing that DCs can play a critical role in eliciting immunity from DNA-based vaccination [14,15].

Chemotactic-tumor associated antigen vaccines have been reported to target specific antigens to special immune cell types, and thereby elicit improved immune responses [16]. Previously it was suggested that the majority of cells transfected by gene gun-mediated vaccination (in skin) were non-antigen presenting cells [17]; however, a relatively small but significant number of transgene-expressing DCs were detected in the draining lymph nodes [18]. Timares and his colleagues [17] also suggested that directly-transfected DCs can incite immune response in a more efficient way than indirectly-transfected DCs. As compared to conventional vaccines, the success of skin vaccination via gene gun delivery has been challenged by its capacity to elicit only limited immunity [19]. In a recent study, we reported that co-vaccination with a chemokine, mouse RANTES (mRANTES) and a tumor-specific antigen gene, human gp100 (hgp100) within a specific time frame can induce a strong cell-mediated immunity against targeted B16/hgp100-tumor cells [20]. Gene gun-mediated bombardment with RANTES cDNA transgene significantly increased CD11c^+^, NK1.1^+^, CD4^+ ^and CD8^+ ^immune cells in test mouse skin. RANTES, which is regulated upon activation of normal T-cell expression and secretion, is a versatile chemokine which can not only recruit leukocytes into the inflamed tissue sites, but can also act as a powerful leukocyte activator [21]. High level induction of RANTES in keratinocytes has been reported for cutaneous wound-repairing [22].

Here, we evaluated the effects of topical application of shikonin to skin to aid trafficking of skin DCs and thus augment the effect of a DNA-based cancer vaccine. We investigated the effects of shikonin on the induction of RANTES in treated skin tissues that subsequently served as the immunization site for hgp100 gene-based cancer vaccines. Our findings suggest that shikonin can effectively enhance the efficacy of the tumor associated antigen-hgp100 DNA vaccine, and its effect is likely mediated via the induction of mRANTES and an increase in the population of skin DCs at the vaccinated site draining into lymph nodes. Possible implication and application of phytochemical shikonin for generic and topical use as an adjuvant in skin-based vaccinations are discussed.

## Methods

### Animals

Male C57BL/6Jnarl mice (6-8-weeks old) were purchased from the National Laboratory Animal Breeding and Research Center, Taipei, Taiwan. All mice were maintained in a laminar airflow cabinet in a room kept at 24 ± 2°C and 40-70% humidity with a 12 h light/dark cycle under specific pathogen-free conditions. All facilities were approved by the Academia Sinica Institutional Animal Care and Utilization Committee, and all animal experiments were conducted under the institutional guidelines established for the Animal Core Facility at Academia Sinica, Taipei.

### Compounds

Shikonin was purchased from Tokyo Chemical Industry. Gallic acid, ferulic acid, curcumin, caffeic acid and epigallocatechin-3-gallate, phorbol ester 12-*O*-tetradecanoylphorbol-13-acetate were purchased from Sigma.

### Tumor cells and transfectants

The B16F10 melanoma cell line, syngeneic in C57BL/6 mice, was purchased from American Type Culture Collection (ATCC). Human gp100-transfected B16 cell line were obtained by stably transfecting B16 cells using the PowderJect gene delivery system (PowderJect Vaccines) as described previously [18,23]. B16/hgp100 cells were maintained in DMEM supplemented with 10% fetal bovine serum, 2 mM L-glutamine, and 100 μg/ml streptomycin and penicillin.

### Construction of cDNA expression vector and transgene

Human gp100 (hgp100) expression plasmid pWRG1644 was a kind gift from Dr Nicholas Restifo, National Cancer Institute, (Bethesda, MD USA). pWRG1644 plasmid without containing the coding sequence of hgp100 was used as a control [24]. The pORF9-mRANTES mammalian expression vector was purchased from Invivogen (San Diego, CA, USA).

### Primer design and reverse transcriptase-PCR condition

Total RNA was extracted as per the manufacturer's instructions of the TRIzol reagent (Invitrogen). RT-PCR reactions were conducted with the AccessQuick RT-PCR system (Promega, Madison, WI), according to the manufacturer's instructions, at cycle numbers that yielded products within the linear range as determined for each specific pair of primers. Specific primer pairs for mouse RANTES were 5'-ATATGGCTCGGACACCACTC and 5'-CCTTCGAGTGACAAACACGA.

### Fluorescein isothiocyanate (FITC) sensitization assay

The assay method was modified from a previous study [25]. FITC was dissolved in acetone/dibutylphthalate (4:1) before application. Mice were painted the abdomen skin with 50 μl of 1% FITC solution. After 30 mins, shikonin (0, 50 or 100 μg/3.8 cm^2^/site) or transgenic bombardments were applied onto the test site. After FITC painting for 24 hours, axillary, brachial and inguinal lymph nodes were obtained, single-cell suspensions were prepared[26], purified by anti-CD11c microbeads, and then stained with PE-anti-MHC class II antibody. Flow cytometry analysis by test cells was performed on a FACScan.

### Mouse models of skin inflammation

Protocols for creating skin inflammation in mice were approved by the Institute Ethical Committee and IACUC. For skin inflammation test, female C57BL/6JNarl mice received a topical application on ~3.8 cm^2 ^area/site of the shaved abdomen with 10 nmol TPA. In selected groups, 50 μl of the vehicle control or solutions of test shikonin concentration was applied on test skin 30 min before challenge. Total RNA was extracted from test skin tissues after 24 hours; chemokine expression was analyzed in subsequent time points by RT-PCR. In each experimental group, some of the mice were sacrificed and the test skin samples were excised for snap frozen for immunohistochemistry or histological analysis.

### Immunohistochemical and histological analysis

Skin tissue samples were collected and fixed in 10% PBS buffered formalin and embedded in paraffin wax. Specimens were cut into 5 μm-thick sections and mounted on slides for the immunohistochemistry by a standard indirect streptavidin-biotin immunoperoxidase staining method. Specimens were treated with primary rat anti-mouse mRANTES, and subsequent secondary biotin-conjugated anti-rat IgG and tertiary peroxidase-conjugated streptavidin (Serotec) [6,27]. Primary antibodies were purchased from PharMingen (San Diego, CA, USA) or Serotec. Apoptotic cells were detected with an *in situ *Cell Death Kit (Roche Diagnostics). All test skin tissue sections were counterstained with hematoxylin.

### The subcutaneous B16/hgp100 tumor model and cytotoxic T lymphocytes (CTL) assay

B16/hgp100 tumor cells were injected subcutaneously (1 × 10^5 ^cells/100 μl/mouse) into the right flank of mice. On day 7 post-tumor cell inoculation, test mice were vaccinated with hgp100 cDNA vector (3 μg each/mice). mRANTES cDNA vector (3 μg each/mice) or shikonin (100 μg/site/mice) was transfected or topically applied to equal area of abdominal skin, 24 hours before the hgp100 cDNA vaccination. One primer and two boosters were administered on day 0, 7 and 14. One week after the last booster, splenocytes and lymph node cells were harvested from immunized mice and assayed for CTL activity assayed according to the manufacturer's protocol (DELFIA, Perkin Elmer, MA, USA). The percentage of specific lysis was calculated as (experimental release (counts)-spontaneous release (counts))/(maximum release (counts) - spontaneous release (counts)) × 100.

### Statistical analysis

ANOVA or the *T*-test statistical method was employed to analyze the data from different groups or treatments of test herbs or phytochemicals.

## Results

### Effect of various phytochemicals on induction of RANTES in mouse skin tissues

**Figure 1:**
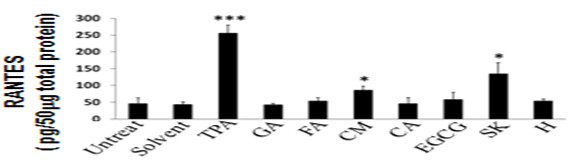
**Induction of mRANTES expression by test phytochemicals in mouse skin tissues**. Mouse skin was either untreated, or treated with vehicle control solvent, gallic acid (GA), ferulic acid (FA), curcumin (CM), caffeic acid (CA), epigallocatechin gallate (EGCG) or shikonin (SK), each at 0.7 mmole/site/mouse. TPA and hydrocortisone (H) were used as a positive and negative control, respectively. Skin samples were collected at 24 h post treatment and mRANTES expression was determined by ELISA analysis. *, *P <*0.05; ***, P <0.001, versus solvent treatment. Error bars indicate SD.

Six anti-inflammatory phytochemicals, gallic acid (GA), ferulic acid (FA), curcumin (CM), caffeic acid (CA), epigallocatechin gallate (EGCG), and shikonin (SK) were selected to evaluate specific phytochemicals that may regulate or modify the expression of mRANTES in skin tissues. The phorbol ester, 12-*O*-tetradecanoylphorbol-13-acetate (TPA), a well-known inflammation-inducing agent, was used as a positive control for induction of mRANTES. A clinically used therapeutic anti-inflammatory drug, hydrocortisone (H), was used as a negative control. H treatment did not affect the expression of mRANTES in mouse skin samples (Figure 1). In comparison, when equal concentrations (each at 0.7 mmole/skin site/3.8 cm^2^/mouse) of the six test phytochemicals were applied topically to mouse skin, two phytochemicals induced expression of mRANTES, c.a., SK and CM. Shikonin induced the highest level of expression of mRANTES protein; close to 3-fold higher than that of the vehicle control. CM also induced mRANTES expression, but to a lesser level than shikonin. In contrast, no significant inductive effect was detected in GA, FA, CA or EGCG-treated skin tissues.

### Shikonin differentially affects expression of mRANTES and MIP-1β mRNAs in normal and inflamed skin tissues

**Figure 2:**
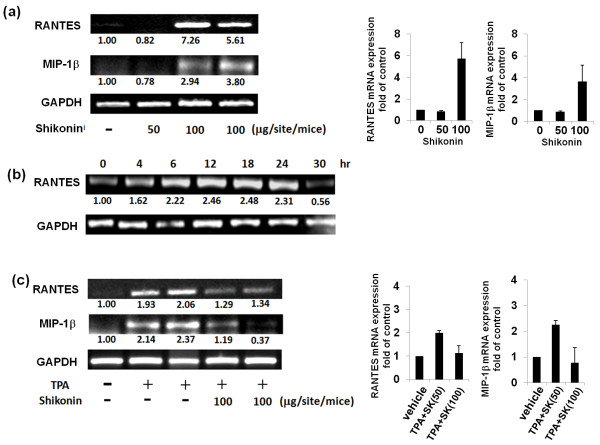
**mRANTES and MIP-1β mRNA expression in non-inflamed and inflamed skins **(a) **Expression of mRANTES induced by different doses of shikonin (50 or 100 μg/site/mice) in non-inflamed skin and statistic analysis data; **(b) **Time course of shikonin-induced mRANTES mRNA expression in non-inflamed skin tissues**. Shikonin was topically applied at 100 μg/site/mice. **(c) **Anti-inflammatory effect of shikonin on the mRNA expression of mRANTES and MIP-1β in a TPA-induced inflamed skin model and statistic analysis data. The results are representative of three independently performed experiments.

After 24 hours of topical treatment of normal skin with two different doses of shikonin, mRNA expression of mRANTES and MIP-1β was assayed. At a dose of 100 μg/site/mouse, shikonin was able to effectively induce mRNA expression of mRANTES and mMIP-1β with approximately 5- to 7-fold and 3- to 4-fold increases, respectively, in comparison to non-treated skin tissue (Figure 2a). These two chemokines have been reported to promote the recruitment of leukocytes which can in turn facilitate the tissue repair process [28,29]. We then assessed the expression pattern of mRANTES mRNA in shikonin-treated skin tissues in a time-course experiment over 36 hours. We observed that an increase in mRANTES mRNA expression was detectable at 4 hours after treatment with shikonin (100 μg/site/mouse); and maximum level of expression was reached between 12 and 18 hours post-treatment (Figure 2b). Interestingly, however, in skin tissue inflamed by TPA, mRANTES and mMIP-1β were markedly downregulated by shikonin (Figure 2c).

### Comparison of shikonin and transgene-induced expression of mRANTES mRNA and protein in skin tissue

**Figure 3:**
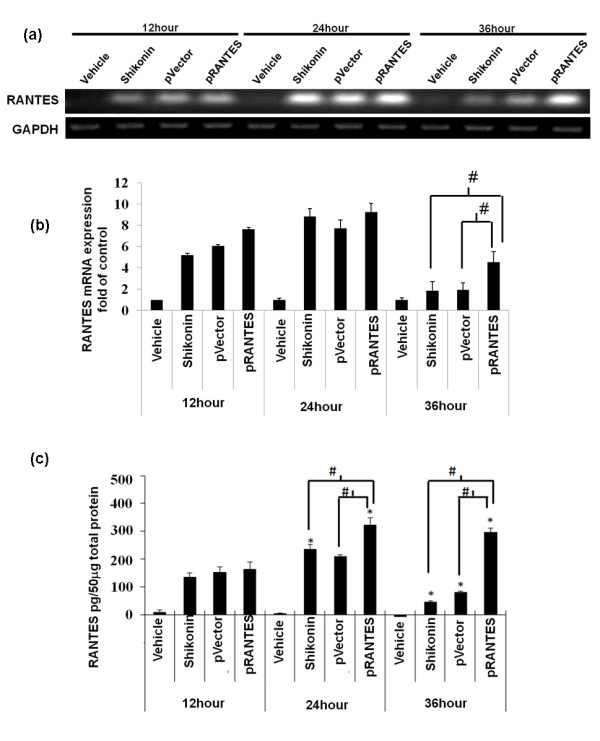
**Comparison of mRANTES expression in skin subjected to topical treatment with shikonin, or gene transfection with mRANTES cDNA expression vector**. Mouse abdominal skin was treated with vehicle or shikonin (100 μg/site/mice), or transfected with an empty vector (pVector) or mRANTES cDNA plasmid (pRANTES) via gene gun bombardment delivery. At the indicated time points, mRNA and protein samples were extracted and expression of mRANTES was analyzed by RT-PCR and ELISA: **(a) **mRNA expression; **(b) **statistic data obtained from **(a)**; **(c) **protein expression.*P <0.05, when compared with indicated vehicle treatment at 12 hours; #, *P <*0.05, between indicated treatments. Error bars indicate SD. The results are representative of three independently performed experiments.

Next, expression levels of mRANTES from shikonin-treated skin tissue were compared with that from mRANTES cDNA vector-transfected skin tissue. The mRNA expression of mRANTES was substantially induced by shikonin at 12 hours post treatment, and the expression was substantially increased at 24 hours post treatment (Figure 3a). The mRNA expression level of RANTES from empty vector-transfection was slightly higher than that obtained with shikonin treatment. Gene gun bombardment is known to mediate protein expression of inflammatory cytokines, which has been reported to result from an intrinsic response to physical stress and tissue damage [8]. RANTES cDNA gene transfection apparently increased the RANTES mRNA and protein expression levels, (Figure 3a and 3c, respectively), over those from the vector only (i.e., tissue wounding only) treated skin tissues. As compared to the "24 hours and 36 hours" -time period for shikonin-induced RANTES expression, the gene gun-mediated transgenic expression at both the mRNA and protein level can reach a substantially higher level, and the expression time frame can last a substantially longer period (Figure 3a and 3c).

### Histological analysis of shikonin- and transgene-induced RANTES protein expression in test skin tissue

**Figure 4:**
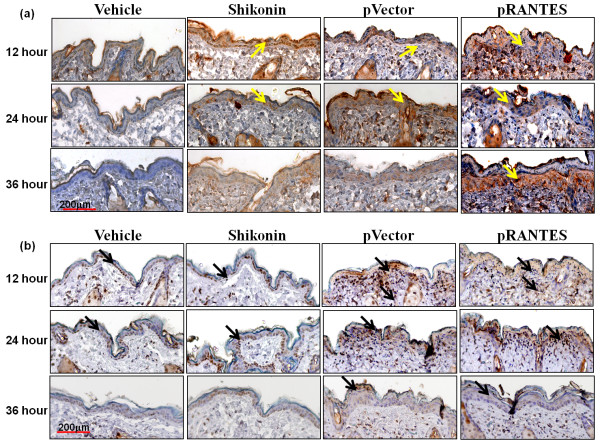
**RANTES expression and level of apoptosis *in situ *in shikonin and mRANTES cDNA transgene-treated skin tissues**. Immunohistochemistry was performed at the indicated time points from vehicle-treated, shikonin-treated, empty pVector-transfected or pRANTES cDNA-transfected skin. **(a) **mRANTES protein expression; brownish staining (yellow arrows) was revealed by immunoperoxidase staining using avidin-biotin-peroxidase complexes counterstained with hematoxylin. **(b) ***In situ *apoptosis detection showing apoptotic cells stained in brownish color (black arrows) was observed in the epidermis. All test skin tissue sections were counterstained with hematoxylin. Similar results were observed in skin biopsies from three different mice, and a representative histological result is shown. Scale bar: 200 μm.

Next we evaluated RANTES expression and regional tissue damage in a time course experiment. To this end, shikonin-treated or RANTES cDNA vector-transfected skin tissues were collected at different time points and subjected to immunohistochemistry assays. RANTES protein expression was mainly detected in the epidermal cell layers (Figure 4a). Vehicle-treated skin expressed little or no RANTES protein. The shikonin induced RANTES expression in the epidermis at 12 hours post treatment gradually decreased between 24 hours and 36 hours post treatment. The pattern of empty DNA vector-transfection resulted expression of RANTES is apparently closely correlated with the physical stress and wound healing activities observed for the bombarded skin tissues, as we have previously reported for gene gun-mediated transfection into skin tissue [20]. In contrast, RANTES cDNA vector-transfection readily induced high level RANTES expression from 12 hours; the expression levels were maintained at 24 hours and were still elevated at 36 hours post gene transfection. It is interesting to note here that the expression of transgenic RANTES test skin was mainly detected in the dermal layer of test skins at 36 hours. This pattern may reflect the transcriptional effects from RANTES transgene activity in skin. This *in situ *RANTES expression pattern at the histological level (Figure 4a) was distinguishable from the results obtained from ELISA assays at total skin tissue level (Figure 3c). A marked increase in level of TUNEL-positive apoptotic cells was detected in gene gun-bombarded skin tissue (pVector or pRANTES) at 12 and 24 hours, and was substantially reduced at 36 hours. When compared to gene gun-bombardment, shikonin treatment induced relatively fewer apoptotic cells in test skin tissue. Together, these results suggest that topical treatment with shikonin may induce mRANTES and increase accumulation of infiltrated cells in test mouse skin tissues.

### Shikonin pre-treatment increases the percentage of skin DCs migration into draining lymph nodes after hgp100 cDNA vaccination

**Figure 5:**
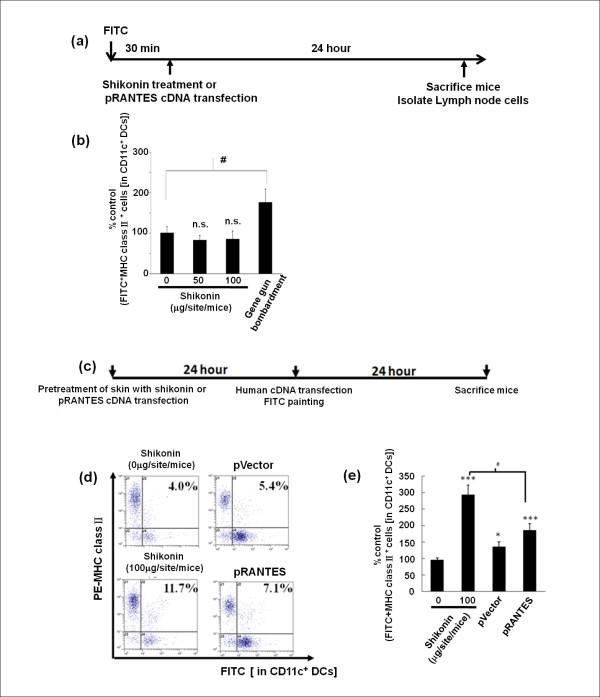
**Shikonin pre-treatment increases level of dendritic-cell migration from skin (immunization site) to draining lymph nodes in hgp100-immunized mice**. **(a) **Scheme for testing DC migration from skin to lymph nodes (LNs). **(b) **Flow cytometry analysis of the the effects of shikonin and transgenic RANTES on DC trafficking in skin. The data represent percentage of control FITC^+^MHC class II^+ ^cells [in CD11c^+ ^DCs]. **(c) **Schema for pretreatment of skin with shikonin or transgenic RANTES and subsequent treatment with hgp100 cDNA vaccination. **(d) **Flow cytometry analysis of DC-trafficking activities in test mice. Mouse abdominal immunization skin tissue sites were pre-treated with shikonin (100 μg/site/mouse) or transfected with RANTES cDNA 24 hours before immunization via gene gun bombardment for transgenic hgp100 expression. **(e) **Flow cytometric analysis of skin DC trafficking activity shown in **(d)**, data represent percentage of control FITC^+^MHC class II^+ ^cells [in CD11c^+ ^DCs]. Error bars indicate SEM. (three mice per group).

Next FITC sensitization assay was performed to trace the trafficking of DCs, especially from the skin vaccination site to draining lymph nodes. A significant increase in CD11c^+^FITC^+^MHC class II^+ ^cells was detected in lymph nodes from mice bombarded with uncoated golden particles (without any cDNA plasmid or the empty vector) when compared to those from shikonin-treated mice (Figure. 5a, b). This data suggests that particle/gene gun-generated physical tissue injury/stress, but not shikonin treatment, can effectively increase (approximately 2 fold) the migration of dendritic cells from skin to lymph nodes *in vivo*. Therefore, we next tested whether topical "shikonin-pretreatment" or "RANTES cDNA transgene-pretransfection" could contribute to an increase in trafficking of DCs from the skin treatment site to the draining lymph nodes, as we postulated that the physical stress resulting from the gene gun treatment could help augment such activities. Interestingly, a significantly higher frequency of CD11c^+^FITC^+^MHC class II^+ ^cells was detected in draining lymph nodes of shikonin-pretreated mice (11.7%), even higher than that in the RANTES transgene-pretransfected mice (7.1%) (Figure 5c, d and 5e).

### Effect of different immunization regimens on specific cytotoxic T lymphocyte activity in test mice

**Figure 6:**
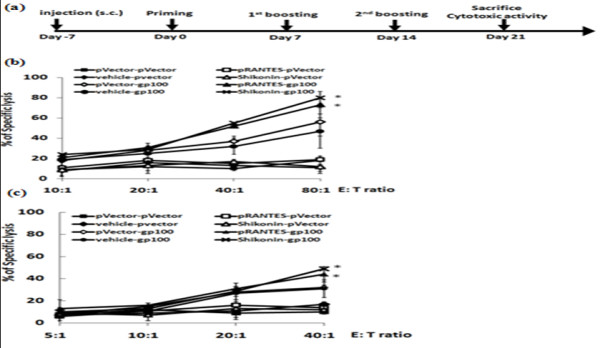
**Specific cytotoxic T lymphcyte activities in test mice treated with different immunization regimes**. **(a) **Schema for gene-based vaccination and adjuvant treatment against test tumors in mouse model. One primer and two boosters were performed on days 0, 7 and 14. Mouse immunization sites were either pre-treated with shikonin (100 μg/site/mice) or transfected with pRANTES vector 24 hours before each primer and booster. The empty vector or vehicle control alone was used as a corresponding control. One week after the last booster, CTL activities from splenocytes **(b) **and lymph node cells **(c) **were determined from immunized mice. Target tumor cells (B16/hgp100) were co-cultured with effector cells at indicated ratios of effector:target (E:T) cells from 5:1 to 80:1 and assayed for CTL activity. *, *P *< 0.05, versus pVector or vehicle controls. (Seven mice per group).

In order to evaluate a possible adjuvantic effect of shikonin on gene gun-delivered hgp100 DNA cancer vaccine onto skin, we compared various different vaccine regimes using combinations of hgp100 DNA vaccine with pre-treatments with shikonin or RANTES cDNA transgene vector in a B16/hgp100 melanoma model. B16/hgp100 tumor-bearing mice were subjected to three hgp100 vaccinations. Twenty-four hours prior to each vaccination, topical application of shikonin or transgenic delivery of RANTES cDNA via gene gun was performed on the skin of test mice at the inoculation site. Among all groups tested, the highest and most potent CTL activities were detected in draining lymph nodes of hgp100 cDNA-immunized mice that were pre-treated with shikonin or pRANTES transgene (Figure 6b, c).

## Discussion

Topical and transdermal delivery of pharmacological agents including vaccines is receiving increased research attention due to recent expansion of the concept of skin to a fascinating immune system. Our previous study showed that a combination of transgenic expression of RANTES and hgp100 as a chemotactic cancer vaccine can elicit a much more efficient immune response than hgp100 cDNA alone [20]. In this study, we first screened several candidate phytochemicals for their possible induction of mRANTES expression in test skin tissue and found that shikonin was able to induce the highest level of mRANTES expression in mouse skin (Figure 1). Shikonin has been shown to exhibit multiple and sometimes seemingly conflicting bio-activities, e.g., anti-tumor vs. wound-healing effects [7,30] and anti-inflammation vs. induction of pro-inflammatory chemokines [3]. A number of studies have also reported that shikonin can exhibit anti-tumor effects via inhibition of cell proliferation [31] and proteasome activity [32], and by circumventing cancer drug resistance via induction of necroptosis [7]. A herbal medicine formulation made from *Lithospermum erythrorhizon *containing shikonin has been shown to enhance granuloma formation [30], and help reconstitution of skin tissue [2] through induction of neovascularization in granulomatous tissue during wound-healing [4]. It is well-known that shikonin confers a potent anti-inflammatory effect, and we have previously shown that this effect on skin may result from inhibition of the promoter activation [8] and mRNA splicing [9] activities for TNFα expression. As a follow up, we showed that shikonin can interfere with or inhibit the maturation of DCs by suppressing certain proinflammatory cytokines. Shikonin treatment substantially affected transgenic and/or endogenous expression of TNF-α, GM-CSF and other cytokine genes in mouse skin tissues *in vivo *[33] and in DCs or monocytes *ex-vivo *[34]. In the present study we have shown that under normal physiological conditions both shikonin and curcumin, which have both been claimed to confer various anti-inflammatory activities, were able to induce expression of mRANTES which is generally considered to be a pro-inflammatory chemokine. Secretion of RANTES by keratinocytes is known to play a role in promoting cutaneous wound-healing mediated via interaction with the TWEAK/Fn14 signaling network [22,35]. This *in situ *RANTES expression pattern at the histological level (Figure 4a) was distinguishable from the results obtained from ELISA assays at the total skin tissues extract level (Figure 3c). Increasing evidences are showing that the immune systems are highly sophisticated and can no longer been dichotomized simply into "pro-inflammation" versus "anti-inflammation" activities. There are overlapping and parallel factors involved in the regulation of both immune responses, as is known for the "multiple facets" of IL-6 [36]. As for the different effects of shikonin under different physiological/pathological microenvironment, together with our own and previous findings from others, we can speculate some possibilities for elucidating these observations. For example, after TPA treatments, several responsive genes can be highly activated, including NFκB, RANTES, MIP-1β and TNFα. Shikonin has been shown and considered as an anti-inflammatory phytochemical, as we have reported previously [8,9]. Therefore, the inhibitory effect of shikonin on TPA-induced RANTES and MIP-1β gene expression may result into such anti-inflammatory activity. On the other hand, in addition to inflammatory signals, the expression of RANTES may be regulated by other signals or factors, such as apoptotic signaling, FasL [37] and calcineurin [38]. The present study indicates that a high dose of shikonin may act as a strong stimulus to the epidermis, which is made up largely of keratinocytes, and induce a high level of RANTES expression, which may partially result from transiently induced apoptotic activities in the epidermis. In comparison with changes seen after gene gun-bombardment of skin, only minor changes in the levels of cell apoptosis in the epidermis and skin damage were seen after shikonin treatment. Although a number of skin vaccination approaches have been explored to boost systemic immunity, new and optimized tissue manipulation techniques for topical vaccination are still needed to address issues such as reducing adjuvant toxicity and disruption of epidermal structure. Within this paradigm, the localized microenvironmental stress induced by shikonin can be considered to be minor. In addition, shikonin treatment did not induce a fast turnover of skin tissues. Overall, the evidence presented here suggests that shikonin may play a "multi-faceted" role in regulating certain activities that induce a diversified repertoire of chemokines expressed in skin, and some of these activities may correlate with the recruitment of DCs.

Several modalities of topical immunotherapy have been developed [39]. A variety of adjuvants with potent immuno-enhancing activities, such as toll-like receptor ligands [40], have been employed for use in skin vaccination [40,41]. RANTES/CCL5 is characterized as a proinflammatory chemokine and can attract CCR5-expressing immune cells, including immature DC and T cells. In our previous study, although we showed that the CD11c^+ ^DCs were significantly increased in mRANTES-transfected skin, we were unable to exclude the possibility that the CD11c^+ ^DCs which were increased in population as a response to treatment with RANTES were immature. The results of this study (Figure 4) and a previous report [19] suggest that a gene gun-mediated DNA vaccine encoding specific tumor-associated transgenes may provide a strong stimulus, due to physical stress and/or a DNA backbone signal which activates skin DCs, that enables DCs fully mature and thus effectively migrate to the draining lymph nodes where T-cell priming can take place. We further suggest that topical application of shikonin can effectively increase immature DC trafficking and accumulation at the immunization site, thus facilitating the effectiveness of subsequent vaccinations.

**Figure 7:**
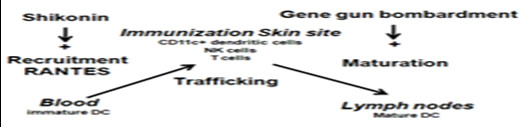
**Proposed mechanism for mode of action of shikonin in skin tissues**. Topical pretreatment with shikonin enhances the recruitment of DCs and other responsive immune cells via induction of RANTES, and in turn, increases the trafficking and population of these immune cells at a specific vaccination site. The increased number of immune cells accumulated at the vaccination site then increases the frequency of exposure or transfection of cells after subsequent DNA-based vaccination. The gene-based vaccines can further provide strong stimuli, due to the gene transfection process or the composition of the DNA vaccines (e.g., CpG), to activate skin DCs with transgenic antigenic signals to migrate to the draining lymph nodes where naïve T cells may be primed.

We further suggest that the induction of mRANTES by shikonin can result in an accumulation of potent antigen presenting cells, CD11^+^DCs and presumably other responsive immune cells such as NK cells, CD4^+ ^and CD8^+ ^T cells, at the treatment target tissue site, which can in turn increase the population of specific antigen presenting cells that are exposed to test antigens by subsequent DNA-based vaccination. An additional gene-based vaccination may further contribute to the potency of the initial vaccine by providing not only the tumor-associated antigenic signals, but also providing a strong stimulus for the maturation of skin DCs. The activated DCs carrying the tumor-associated antigenic signals may then migrate to the draining lymph nodes and elicit targeted innate and adaptive immunities (Figure 7). An intriguing finding obtained from this study is that shikonin, which has so far mainly been recognized as a potent anti-inflammatory agent, can also induce potent expression of the pro-inflammatory chemokine mRANTES in un-inflamed or un-stimulated skin tissues. The differential effects of shikonin on skin in different physiological environments may result from an interaction between shikonin and different participating or neighboring cells and/or the intrinsic chemical nature of shikonin which can modulate a specific balance between the pro- and anti- inflammatory cytokines and chemokines[42,43]. In this regard, our present results showed that a specific immunization regime (i.e., shikonin pretreatment/hgp100 cDNA gene vaccination) increased the population of migrated skin DCs in draining lymph nodes (Figure 4) and augmented CTL activities (Figure 6) in vaccinated mice. These results also show that the anti-inflammatory activity of shikonin may only have a minor restraining effect on the cellular events generated by the subsequent vaccination.

## Conclusion

In conclusion, our present study investigated the mechanistic and multi-targeted molecular effect of shikonin on the trafficking of DCs, and we evaluated its potential application as a DNA vaccine adjuvant. We showed that shikonin, a phytochemical purified from *Lithospermum erythrorhizon*, can induce the highest level of expression of RANTES in normal skin tissues among a number of anti-inflammatory phytochemicals tested. In comparison with mouse RANTES cDNA gene transfection, shikonin treatment induced a transient high-level expression of mRANTES (up to 24 hours) and this caused less extensive skin tissue damage. A human gp100 cDNA gene vaccination regimen in combination with pretreatment with shikonin as an adjuvant in a human gp100-transfected B16 mouse tumor model significantly increased the cytotoxic T lymphocyte activities on target tumor cells in splenocytes and lymph node cells of the test mice. We believe this shikonin treatment approach may be beneficial/promising for future clinical application to gene-based cancer vaccines. Future optimization of a DNA vaccination schema and regimen may include the efforts to reduce the undesirable effects of shikonin and amplify its beneficial effect. Shikonin, as a natural phytochemical product, warrants further evaluation as an adjuvant for gene-based vaccines, especially for topical application alongside vaccination or immunotherapy. The present study may serve as a pilot approach or system for evaluating the use of an immuno-stimulatory agent (e.g., shikonin) as an adjuvant for topical application on skin alongside vaccines and gene delivery vectors.

## Abbreviations

DC: dendritic cells; RANTES: regulated upon activation of normal T-cell expression and secretion; Hgp100: human gp100; FITC: fluorescein isothiocyanate; GA: gallic acid; FA: ferulic acid; CM: curcumin; CA: caffeic acid; EGCG: epigallocatechin gallate; SK: shikonin; CTL: cytotoxic T lymphocytes.

## Competing interests

The authors declare that they have no competing interests.

## Authors' contributions

HMC conceived of the study, and participated in its design and coordination and helped to draft the manuscript. PSW conducted immunohistochemistry assay. KA designed the PCR primers for mouse RANTES and MIP-1β. WCY was the consultant for this manuscript. YHC and HHY were responsible for the preparation of gene gun experiments. All authors read and approved the manuscript.
